# Psychological resilience mediates the protective role of default-mode network functional connectivity against COVID-19 vicarious traumatization

**DOI:** 10.1038/s41398-023-02525-z

**Published:** 2023-06-29

**Authors:** Xiqin Liu, Yajun Zhao, Xueling Suo, Xun Zhang, Nanfang Pan, Graham J. Kemp, Qiyong Gong, Song Wang

**Affiliations:** 1 Department of Radiology and Huaxi MR Research Center (HMRRC), Functional and Molecular Imaging Key Laboratory of Sichuan Province, West China Hospital, Sichuan University, Chengdu, China; 2 School of Education and Psychology, Southwest Minzu University, Chengdu, China; 3 Liverpool Magnetic Resonance Imaging Centre (LiMRIC) and Institute of Life Course and Medical Sciences, University of Liverpool, Liverpool, UK

**Keywords:** Predictive markers, Human behaviour

## Abstract

Vicarious traumatization (VT), a negative reaction to witnessing others’ trauma, has been experienced by some people during the COVID-19 pandemic, and can lead to mental health problems. This study aimed to identify functional brain markers of COVID-specific VT and explore the psychological mechanism underlying the brain-VT link. One hundred healthy participants underwent resting-state functional magnetic resonance imaging before the pandemic (October 2019–January 2020) and completed VT measurement during the pandemic (February–April 2020). Whole-brain correlation analysis based on global functional connectivity density (FCD) mapping revealed that VT was negatively correlated with FCD in the right inferior temporal gyrus (ITG) (i.e., the lower FCD in ITG, the worse the VT), identified by mapping onto known large-scale networks as part of the default-mode network (DMN). Resting-state functional connectivity (RSFC) analysis using ITG as seed found that VT was predicted by lower functional connectivity between ITG and other DMN regions including left medial prefrontal cortex, left orbitofrontal cortex, right superior frontal gyrus, right inferior parietal lobule and bilateral precuneus (i.e., the lower the ITG-DMN connectivity, the worse the VT). Mediation analyses suggested that psychological resilience served as a mediator in these associations of ITG FCD and ITG-DMN RSFC with VT. Our results provide novel evidence on the brain basis of VT and emphasize psychological resilience as an important link from DMN functional connectivity to COVID-specific-VT. This may facilitate public health interventions by helping identify individuals at risk of stress- and trauma-related psychopathologies.

## Introduction

The Coronavirus Disease 2019 (COVID-19) pandemic has caused massive disruptions to our lives and health [1, 2]. Its rapid spread and the worldwide lockdowns have increased psychological stress in the face of uncertain and uncontrollable threats [3, 4]. People in social isolation rely more on social media and the internet for information [5, 6], which can help alleviate anxiety. However, repeated and excessive media exposure may lead to vicarious traumatization (VT) [4, 5, 7], defined as the repetitive invasion of another’s traumatic experiences [8, 9]. VT is typified by stress, burnout, fatigue, loss of confidence and decreased well-being, and in different populations can lead to anxiety, depression and posttraumatic stress disorder (PTSD) symptoms [10, 11]. The association between COVID-19 and VT has been established [5, 7, 12], but the underlying neurobiology remains largely unknown. Elucidating how individual differences in brain function impact VT could help to identify individuals vulnerable to stress- and trauma-related psychopathologies, and thus provide intervention targets. Our first aim was therefore to define prospective functional neural markers of COVID-related VT.

VT varies with situational and personal factors such as socioeconomic status, previous trauma history, coping strategies, the ability to tolerate strong affect, and psychological resilience [13–15]. Psychological resilience, defined as the ability to adapt positively to difficulties and recover from significant adversity, trauma, or stressful events [16], is particularly important in determining whether an individual develops PTSD after exposure to traumatic events [17]. Resilience also has a protective role in vicarious trauma, reducing anxiety and depression symptoms and helping recovery [15]. Psychological resilience is negatively associated with COVID-19-related fear, worry, stress, anxiety, depression and negative affect [18], which are major symptoms of VT [10], suggesting that the risk and severity of COVID-related VT might be enhanced by lack of psychological resilience. Thus, our second aim was to examine whether psychological resilience might be a mechanism through which brain function influences VT, i.e., an intervening variable in the brain-VT association.

There is abundant neuroimaging evidence that stress and stress-related disorders such as PTSD are mediated by brain systems, notably including the limbic regions (e.g., hippocampus and amygdala) and prefrontal cortex [19, 20], which belong to the default mode network (DMN) [21]. It is increasingly recognized that higher-order cognitive and emotional functions (important in acute and chronic stress) and stress-related psychopathology depend on distributed large-scale brain networks, not isolated regions [22, 23]. Functional magnetic resonance imaging (fMRI) studies, both task-based and resting-state, highlight the pivotal role of DMN in normal and pathological stress [20, 24–26]. For example, previous studies have reported altered intrinsic functional activity and connectivity and volumetric reduction of the DMN in healthy subjects with higher perceived stress [27, 28]. Also, altered intrinsic connectivity of DMN has been associated with PTSD patients [29, 30], PTSD symptoms [31], mood disorders [32] and the severity of anxiety [33], suggesting escalating episodic thoughts and emotion processing [34, 35]. In the opposite sense, increased volume in DMN regions (e.g., medial prefrontal cortex, hippocampus) [36, 37] and DMN integration have been linked to psychological resilience [32, 38]. These findings suggest a potential mechanism whereby DMN may underpin the individual variations in VT, with psychological resilience serving as a mediator.

This prospective study exploited pre-pandemic resting-state fMRI (RS-fMRI) data to predict VT during the pandemic in a group of normal university students. First, we used voxel-level functional connectivity density (FCD) mapping to identify the functional brain markers of VT through whole-brain correlation analysis. FCD is a sensitive and reproducible data-driven method of identifying brain ‘hubs’ by analyzing whole-brain functional connectivity patterns at the voxel level: a voxel’s FCD value measures its functional connectivity to other voxels, and thus its putative importance in information processing [39, 40]. FCD mapping has been used to identify abnormal functional hubs in neuropsychiatric disorders [41, 42], and the neural correlates of behavioral constructs in healthy populations [43, 44]. Next, we performed resting-state functional connectivity (RSFC) analysis to explore specific functional couplings with the cluster(s) identified from the FCD-behavior correlation analysis, and to test their ability to predict VT. Then we used correlation analysis and meditation analysis to explore the potential role of psychological resilience in linking pre-pandemic FCD and RSFC with COVID-specific VT. Based on previous findings, we hypothesized that pre-pandemic FCD and RSFC in DMN regions would predict the levels of VT during the pandemic, and that psychological resilience would mediate the brain-VT associations.

## Materials and methods

### Participants

A total of 151 healthy, right-handed Chinese university students (74/77 male/female, age 19–27 years), reporting no history of psychiatric or neurological diseases, participated in this study, which was part of a large ongoing project investigating the relation between brain and mental health [25, 45–47]. All 151 participants underwent MRI scanning and completed paper-based questionnaires between October 2019 and January 2020 (T1, prior to the declaration of emergency state and city lockdown in China due to COVID-19). They were then re-contacted and invited to take a COVID-19-related behavioral online survey between February and April 2020 (T2, the pandemic initial outbreak and peak period in China), and 115 participants provided valid responses at T2. After excluding 15 participants with excessive head motion (see *Image pre-processing*), 100 participants (42/58 male/female, age 19–27 years) contributed to data for the final analyses. This sample size is sufficient to obtain medium-to-large effects for correlation analyses, by standard power analysis [48]. Notably, none of the participants were infected with COVID-19, proved by negative nucleic acid tests. Both behavioral and MRI protocols were approved by the Medical Research Ethics Committee of West China Hospital of Sichuan University. We obtained written informed consent from each participant before the study.

### Behavioral measurements

#### Vicarious traumatization questionnaire (VTQ)

VT was assessed with a 38-item scale originally developed for investigating trauma helpers in the 2008 Sichuan earthquake [49]. The VTQ measures two facets: physiological responses (11 items) and psychological responses (27 items), the latter including cognitive responses (5 items), behavioral responses (7 items), emotional responses (9 items) and life belief (6 items). At T2, participants were asked to rate how often they felt a certain way after the COVID-19 pandemic on a five-point Likert scale from 1 (never) to 5 (always). The total VTQ score was calculated by summing the responses for each item (ranging from 38 to 190), a higher score indicating worse VT. VTQ has adequate reliability and validity [49, 50], and has been used to assess COVID-related VT in professional and general public populations [12, 25]; its Cronbach’s α in this study was 0.95, indicating excellent internal reliability.

#### Connor–Davidson resilience scale (CD-RISC)

Psychological resilience was measured at both T1 and T2 by the Chinese version of the 10-item CD-RISC [51], a widely-used measure of psychological resilience [52]. Each item was rated by participants on a five-point Likert scale from 1 (strongly disagree) to 5 (strongly agree). The total CD-RISC score was calculated by summing the responses for each item (ranging from 10 to 50), a higher score reflecting greater psychological resilience; its Cronbach’s α in this study was 0.83 at T1 and 0.88 at T2, indicating adequate internal reliability. We used the mean CD-RISC score from T1 and T2 as the index of psychological resilience, because there were no significant differences in CD-RISC score from T1 and T2, and the scores at T1 and T2 were highly correlated (*r* = 0.65, *p* < 0.001), suggesting that psychological resilience is a stable personality trait.

#### Other controlling measures

Several controlling measures at T1 were used to exclude potential confounding effects on the links between VT, psychological resilience and functional connectivity. These included the Socioeconomic Status Scale (SSS), which assesses individuals’ family socioeconomic status (SES) [53] and the Self-Rating Life Events Checklist (SRLEC), which assesses the frequency and impact of stressful life events over the past 12 months [54]; their Cronbach’s α in this study was 0.76 (SSS) and 0.91 (SRLEC), indicating adequate internal reliability.

### MRI scanning and preprocessing

#### MRI scanning

MRI data were collected on a 3.0T Siemens-Trio Erlangen MRI system with a 12-channel head coil at West China Hospital of Sichuan University. The RS-fMRI image data were acquired using a gradient-recalled echo-planar imaging sequence: 240 volumes, echo time (TE) 30 ms, repetition time (TR) 2000 ms, 30 slices, voxel size 3.75 × 3.75 × 5 mm^3^, thickness 5 mm, field of view 24 × 24 cm^2^, matrix 64 × 64, flip angle 90°. During the scanning, participants were asked to lie still with eyes closed and not to think of anything particular or fall asleep. High-resolution T1-weighted anatomical MRI images were additionally obtained to improve normalization of the functional images (TR 1900 ms, TE 2.26 ms, flip angle 9°, 176 slices, voxel size 1 × 1 × 1 mm^3^, matrix 256 × 256).

#### Image pre-processing

The RS-fMRI images were preprocessed using DPABI software [55] in the following steps: removing the first 10 images, slice-timing correction, realignment, co-registration of functional and structural images, normalization with the Diffeomorphic Anatomical Registration Through Exponentiated Lie (DARTEL) strategy [56], resampling to 3 × 3 × 3 mm^3^ isotropic voxels, spatial smoothing with a 6 mm full-width half-maximum, linear trend removal, and temporal filtering at 0.01–0.08 Hz. White matter, cerebrospinal fluid signals and head motion parameters were regressed out as nuisance covariates. The mean framewise displacement (FD) of each participant was calculated and participants with excessive head motion (mean FD > 0.25 mm) were excluded from analyses, leading to a final sample of 100 participants. Motion scrubbing was applied for the final sample based on the FD threshold of 0.50 mm [55]; on average, 16 frames were scrubbed per subject (mean percentage = 6.99%, standard deviation = 5.77%).

#### FCD calculation

The pre-processed RS-fMRI data were used to compute the FCD map for each participant using DPABI software [55]. We first calculated the Pearson’s correlation coefficients between the time series of each pair of voxels across the brain, obtaining a whole-brain functional connectivity matrix. To avoid counting the voxels with weak temporal correlations due to signal noise, a threshold of 0.6 was applied to each correlation coefficient in the matrix: a correlation > 0.7 would lead to lower sensitivity, while a correlation < 0.4 would increase false-positive rates for the FCD maps [39, 40]; 0.6 has been the most widely used and reliable threshold to detect brain functional modules [39, 40]. Next, the binary FCD of a voxel was calculated as the number of significant suprathreshold correlations between a given voxel and all other voxels. Finally, the grand mean scaling was applied to the voxel-wise FCD map for each participant by dividing the FCD of each voxel by the mean value of all brain voxels to increase the normality [57, 58]. Particularly, the normality tests revealed that the FCDs in the whole-brain and each major resting-state functional network were normally distributed (Supplementary Table S1), suggesting the scaled FCD maps were suitable for parametric statistical analyses.

### Statistical analyses

#### Whole-brain FCD-behavior correlation analysis

To identify the brain regions in which the FCD was related to COVID-related VT, we performed a whole-brain correlation analysis between the VTQ scores and voxelwise FCD values, with age, sex and FD as the nuisance covariates. In addition, to investigate sex differences in the link between VT and FCD, a condition-by-covariate interaction analysis [46, 59] was performed with sex as a condition, the scores of VT as covariates of interest, and age and FD as confounding covariates. The resulting map was corrected for multiple comparisons with a cluster-level threshold of *p* < 0.05 combined with a voxel-level threshold of *p* < 0.001 using Gaussian random field (GRF) theory [60], which takes both spatial extent and peaks into account by modeling noise as Gaussian random fields [46, 61]. These analyses were conducted using REST software [62].

#### RSFC-behavior correlation analysis

We used this to explore to what extent the region identified in the FCD-behavior correlation analyses interacts with other regions to account for variation in the VT. We used the significant cluster linked with VTQ as the seed regions of interest (ROI). For each participant, a mean time series for the seed region was initially computed and then correlated with the time series of other voxels in the brain. A correlation map was produced for the seed. For the purpose of standardization, the raw correlation map was normalized to a *z-*score map by the Fisher’s *r*-to-*z* transformation. In the group-level analysis, we correlated the VTQ scores with voxelwise RSFC values in the *z*-score map, with age, sex and FD as the nuisance covariates. Significance threshold for the resulting map was set at *p* < 0.05 at cluster level and *p* < 0.001 at voxel level based on GRF theory [46, 60, 61]. These analyses were conducted using REST software [62].

#### Prediction analysis

To examine the robustness of the brain-VT linkage and to avoid overfitting and potential effect of factors such as data distribution and outliers, we implemented a machine learning approach based on four-fold balanced cross-validation using linear regression [46, 63–66]. The whole dataset was randomly and equally divided into fourfolds with a balanced variable distribution. For each of the fourfolds, a linear regression model with FCD or mean RSFC values of the identified cluster as the independent variable and VTQ scores as the dependent variable was estimated using data from the other three folds as predictors. After data from all fourfolds had been predicted, the correlation between the predicted and observed values [i.e., *r*_(predicted, observed)_] was calculated. This procedure was repeated four times and the mean *r*_(predicted, observed)_ was obtained to measure the overall prediction performance. According to established statistical significance testing [63–67], a nonparametric randomization approach was implemented by generating 5000 surrogate datasets of final *r*_(predicted, observed)_ to estimate the null distribution to infer the significance. Age, sex and FD were regressed out before the prediction analyses. These analyses were performed in Matlab R2010a (The MathWorks, Inc., Natick, MA) with the codes used in our previous studies [67–69].

#### Mapping onto large-scale brain networks

To characterize the clusters identified from FCD/RSFC-behavior correlation analyses at a large-scale network level, we overlaid them onto 7 core networks [70]: default mode network [DMN], dorsal attention network [DAN], central executive network [CEN], affective network [AFN], somatomotor network [SMN], ventral attention network [VAN], and visual network [VN]; for details see https://surfer.nmr.mgh.harvard.edu/fswiki/CorticalParcellation_Yeo2011. We evaluated the relative distribution (i.e., the number of overlapping voxels within a network divided by the identified cluster) to measure the similarity of the identified region to the large-scale networks [47].

#### Mediation analyses

To explore the indirect effect of FCD or RSFC on VT through psychological resilience, we used the PROCESS macro in SPSS [70] to build two mediation models. In the two mediation models, FCD or mean RSFC of the identified clusters was treated as the predictor variable (X), the mean CD-RISC score for T1 and T2 was treated as the mediator variable (M), and VTQ score was treated as the outcome variable (Y), with age, sex and FD as the controlling variables. In the mediation model, *path a* is the X-M relation, *path b* is the M-Y relation after adjusting for the X, *path c* is the X-Y relation, and *path c’* is the X-Y relation after adjusting for the M. The indirect effect of X on Y through M can be tested through the significance of *c - c’* from a bootstrap test (5000 trials). An empirical 95% confidence interval that did not contain 0 signified that the indirect effect was significant at *p* < 0.05 [69].

## Results

### Descriptive statistics and bivariate correlations of study measures

These are presented in Table 1. VT at T2 was negatively correlated with psychological resilience measured as the mean score of CD-RISC at T1 and T2 (*r* = −0.32, *p* < 0.001) (also separately with CD-RISC score at T1 [*r* = −0.26, *p* = 0.008] and at T2 [*r* = −0.33, *p* < 0.001]; Table 1); the association remained significant after adjusting for age, sex and FD (*r* = −0.31, *p* = 0.002). VT was not correlated with age (*r* = 0.01, *p* = 0.944), sex (*r* = 0.11, *p* = 0.287) or FD (*r* = −0.13, *p* = 0.197).

**Table 1:** Means, SDs, ranges and bivariate correlations of study variables (*N* = 100).

Variable (Time)	Mean ± SD	Range	1	2	3	4	5	6	7	8
1. Sex^a^ (T1)	–	–	–							
2. Age (years) (T1)	22.43 ± 2.12	19–27	−0.09	–						
3. FD (mm) (T1)	0.16 ± 0.04	0.05–0.24	−0.11	−0.08	–					
4. Family SES (T1)	9.88 ± 2.98	3–18	−0.01	0.03	−0.08	–				
5. SRLEC-Number (T1)	12.73 ± 5.78	1–27	−0.07	−0.04	0.09	−0.23^*^	–			
6. SRLEC-Impact (T1)	29.37 ± 16.95	2–77	0.03	−0.01	0.06	−0.23^*^	0.92^***^	–		
7. CD-RISC (T1)	36.81 ± 4.96	25–48	−0.14	0.01	0.11	0.18	−0.05	−0.10	–	
8. CD-RISC (T2)	35.03 ± 5.80	20–48	−0.15	−0.04	0.06	0.23^*^	−0.04	−0.06	0.65^***^	–
9. VTQ (T2)	74.56 ± 22.99	38–128	0.11	0.01	−0.13	−0.23^*^	0.15	0.25^*^	−0.26^**^	−0.33^***^

*N* number, *SD* standard deviation, *FD* framewise displacement, *SES* socioeconomic status, *SRLEC* Self-Rating Life Events Checklist, *CD-RISC* Connor–Davidson Resilience Scale, *VTQ* Vicarious Traumatization Questionnaire. Timepoints: T1, October 2019–January 2020; T2, February–April 2020.

***p < 0.001; **p < 0.01; *p < 0.05.

^a^Male, 0; Female, 1.

### FCD analyses

**Fig. 1:**
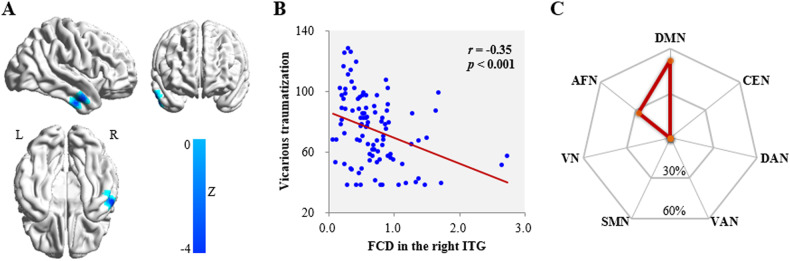
Functional connectivity density (FCD) linked with vicarious traumatization. **A** Brain images showing that vicarious traumatization is negatively linked to FCD in the right ITG after adjusting for sex, age and head motion (color key indicates the strength of negative correlation). **B** Scatter plot depicting the correlation between vicarious traumatization and FCD in the right ITG. **C** Plot showing the similarity of co-activation pattern of right ITG to large-scale functional networks. L left, R right, ITG inferior temporal gyrus, DMN default mode network, CEN central executive network, DAN dorsal attention network, VAN ventral attention network, SMN somatomotor network, VN visual network, AFN affective network.

Whole-brain FCD-behavior correlation analyses found only one significant relation between VT and FCD in the right inferior temporal gyrus (ITG) (*r* = −0.35, *p* < 0.001; Table 2, Fig. 1A, B). Moreover, the condition-by-covariate interaction analysis found no significant regions for an interaction effect of sex by VT.

**Table 2:** Brain regions where FCD and RSFC linked with vicarious traumatization.

Region	BA	Peak MNI coordinate			Peak Z	Cluster size (mm^3^)
		x	y	z	Score	
Correlation with FCD						
R ITG	20/21	63	−6	−30	−3.88	1809
Correlation with RSFC (R ITG as the seed)						
L MPFC	32/10	−12	42	6	−3.83	1620
L OFC	11/10	−30	60	−12	−4.23	1539
R SFG	6/8	18	21	60	−4.02	1053
R IPL	39/40	60	−54	21	−3.66	1809
Precuneus	7/31	18	−57	30	−4.12	6831

*FCD* functional connectivity density, *RSFC* resting-state functional connectivity, *BA* Brodmann’s area, *MNI* Montreal Neurological Institute, *R* right, *L* left, *ITG* inferior temporal gyrus, *MPFC* medial prefrontal cortex, *OFC* orbitofrontal cortex, *SFG* superior frontal gyrus, *IPL* inferior parietal lobule.

Based on the relationship in Fig. 1B, prediction analyses showed that FCD in the right ITG can significantly predict VT across individuals (*r*_[predicted, observed]_ = 0.33, *p* < 0.001) after adjusting for sex, age and FD.

Mapping the ITG cluster identified in the FCD-behavior correlation analyses onto the large-scale intrinsic functional connectivity atlas [70] (Fig. 1C), the majority of voxels were in the DMN (relative distribution [RD]: 52.5%), followed by AFN (RD: 27.5%).

### RSFC analyses

**Fig. 2:**
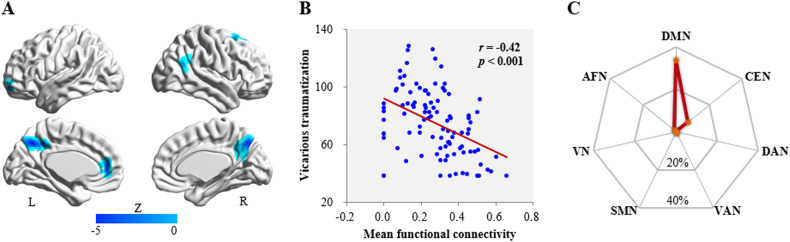
Resting-state functional connectivity (RSFC) linked with vicarious traumatization. **A** Brain regions whose functional connectivity strengths with the right ITG (seed) are linked to vicarious traumatization: left medial prefrontal cortex, left orbitofrontal cortex, right superior frontal gyrus, right inferior parietal lobule and bilateral precuneus. **B** Scatter plot showing the correlation between vicarious traumatization and the overall mean functional connectivity strength of these brain regions with right ITG. **C** Similarity of co-activation pattern of brain regions linked with right ITG to large-scale functional networks. L left, R right, ITG inferior temporal gyrus, DMN default mode network, CEN central executive network, DAN dorsal attention network, VAN ventral attention network, SMN somatomotor network, VN visual network, AFN affective network.

Using the ITG cluster identified in the FCD-behavior correlation analysis as the seed region to explore interactions with other regions, VT was negatively associated with the RSFC between right ITG and 5 regions: left medial prefrontal cortex (MPFC), left orbitofrontal cortex (OFC), right superior frontal gyrus (SFG), right inferior parietal lobule (IPL) and bilateral precuneus (Table 2 and Fig. 2A). Figure 2B shows the significant correlation between VT and the overall mean RSFC between right ITG and these 5 brain regions (*r* = −0.42, *p* < 0.001). Supplementary Fig. S1 shows the significant correlations between VT and the RSFC of ITG with each of the 5 regions.

Based on the relationship in Fig. 2B, VT could be significantly predicted across individuals by the mean RSFC between right ITG and the identified regions (*r*_[predicted, observed]_ = 0.39, *p* < 0.001) after adjusting for sex, age and FD.

Mapping these functional connected regions onto the large-scale intrinsic functional connectivity atlas [70] (Fig. 2C), the majority were in DMN (RD: 33.6%) with a small portion in CEN (RD: 7.4%) and AFN (RD: 1.3%). In what follows we therefore refer to this seed-based RSFC as ‘ITG-DMN connectivity’.

### Mediator role of psychological resilience

To test our hypothesis that psychological resilience may mediate the brain-VT link, we first examined the associations between psychological resilience and the ITG FCD or ITG-DMN connectivity that had been shown to be correlated with VT. As expected, psychological resilience was positively related to FCD in ITG (*r* = 0.24, *p* = 0.02) and ITG-DMN connectivity (*r* = 0.30, *p* = 0.003) after adjusting for age, sex and FD.

**Fig. 3:**
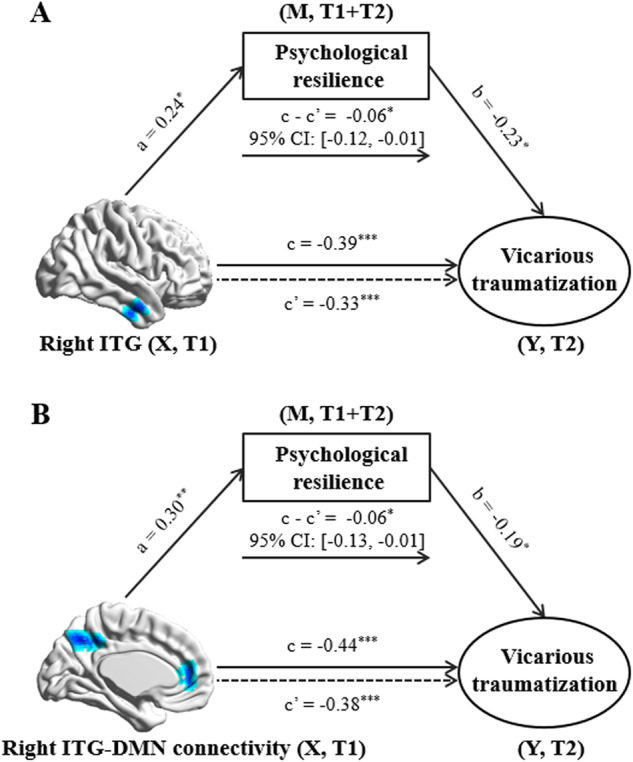
Mediator role of psychological resilience in the association of FCD and RSFC with vicarious traumatization. Psychological resilience mediates the linkage of (**A**) right ITG FCD and (**B**) right ITG-DMN connectivity to vicarious traumatization. Sex, age and head motion are controlled for and standardized estimates are indicated in the path diagram (***p < 0.001; **p < 0.01; *p < 0.05). ITG inferior temporal gyrus, DMN default mode network, X independent variable, M mediator variable, Y dependent variable, CI confidence interval, T1 October 2019–January 2020, T2 February–April 2020.

These results demonstrate that psychological resilience, VT and brain connectivity are closely linked. To explore the nature of the links, we performed mediation analyses while controlling sex, age and FD. The effect size of the negative association between FCD of the right ITG and VT (*c* = −0.39, *p* < 0.001) decreased after including psychological resilience as an intervening variable in the model (*c’* = −0.33, *p* < 0.001). Bootstrap simulation (*n* = 5000) further confirmed the significant mediation effect of psychological resilience on the relationship between FCD of the right ITG and VT (indirect effect = −0.06, 95% CI = [−0.12, −0.01], *p* < 0.05; Fig. 3A). Similarly, psychological resilience had a significant mediation effect on the relationship between ITG-DMN connectivity and VT (indirect effect = −0.06, 95% CI [−0.13, −0.01], *p* < 0.05; Fig. 3B). Thus, psychological resilience partially mediates the effect of functional connectivity on COVID-related VT.

### Specificity of findings

We interrogated our main analyses by adding SSS and SRLEC scores as additional confounding variables. The results proved robust: after controlling for SSS and SRLEC as well as age, sex and FD, VT was still significantly linked with psychological resilience (*r* = −0.26, *p* = 0.01), FCD in the ITG (*r* = −0.37, *p* < 0.001) and ITG-DMN connectivity (*r* = −0.45, *p* < 0.001); and there was still a significant mediation effect of psychological resilience on the linkage of VT to FCD in the ITG (indirect effect = −0.04, 95% CI = [−0.10, −0.01], *p* < 0.05) and to ITG-DMN connectivity (indirect effect = −0.04, 95% CI = [−0.09, −0.01], *p* < 0.05).

## Discussion

In this prospective study of young healthy adults, we identified brain functional connectivity markers of COVID-related VT using FCD and RSFC analyses based on RS-fMRI. There are two key findings: (1) higher VT was predicted by lower FCD in the right ITG (which belongs to the DMN), and by lower functional coupling between ITG and other DMN regions including left MPFC, left OFC, right SFG, right IPL and precuneus; (2) psychological resilience mediated the effect of ITG FCD and ITG-DMN RSFC on VT. These findings suggest potential neurobiological markers for susceptibility to COVID-related VT, and highlight the psychophysiological role of pre-pandemic brain functional connectivity and psychological resilience.

Individual VT scores were predicted by FCD in the right ITG and RSFC of ITG-DMN. There is increasing evidence for the importance of ITG and DMN in the pathophysiology of stress-related mental disorders and emotional dysfunction [71, 72]: structural and functional deficits in ITG have been reported in PTSD [73], bipolar disorder [74], major depressive disorder (MDD) [75] and anxiety disorders [76, 77]; the RSFC of right ITG with other regions in DMN is associated with the severity of anxiety [33], PTSD [30, 78] and mood disorders [74, 79]; DMN RSFC is related to PTSD symptoms [31]; and in task-based fMRI studies brain activity in DMN regions is associated with burnout severity [80, 81]. The core regions of DMN are MPFC, posterior cingulate/precuneus, IPL, ITG and hippocampal formation [21]. ITG, on the lateral and inferior surface of the temporal cortex, is a key node of DMN [82, 83]. With widespread connections to cortical (e.g., middle frontal gyrus, orbital gyrus, precuneus, fusiform gyrus, and middle temporal gyrus) and subcortical areas (e.g., parahippocampal gyrus and hippocampus), ITG is involved in high-order cognitive functions including visual recognition, visual mental imagery, visual semantic memory and language comprehension [84]. ITG and DMN regions also contribute to social-emotion functions such as self-referential processing [85], discrete emotion representation [34], empathic care and distress [86], and mentalizing and vicariously sharing others’ internal states [87]. Altogether, decreased FCD in ITG and ITG-DMN connectivity in this study might reflect impaired internally focused thought and empathetical engagement that contributed to high COVID-related VT.

Note that the ITG identified from the whole-brain FCD-behavior correlation analysis was also mapped onto the AFN, which is primarily associated with emotional processing, memory and motivation [88]. This finding is consistent with previous studies showing dysfunction of the AFN in trauma and PTSD [89, 90]. Similarly, regions from the RSFC-behavior correlation analysis were also mapped onto the CEN. Prior studies have observed decreased RSFC in certain CEN regions (e.g., MPFC) in PTSD [91, 92]. Our study found decreased RSFC between ITG and CEN regions in predicting VT, indicating that cognitive deficits and poor top-down emotional control might underlie VT.

We found significant negative correlation between VT and psychological resilience, consistent with behavioral evidence of the protective role of psychological resilience against symptoms of VT [15, 93] and adverse COVID effects [18, 94]. For the first time our study provides prospective confirmation of psychological resilience-VT association during the pandemic, its specificity and robustness attested by preservation even after adjustment for pre-pandemic family SES and other stressful life events. This supports the concept that resilience can protect those exposed to traumatic events from developing PTSD [17], underlying the well-known variability in responses to traumatic events [95] or childhood adversity [96].

Importantly, psychological resilience mediated the effect of DMN connectivity on VT. Other studies in healthy subjects [97, 98] have also found correlations between psychological resilience and DMN connectivity: for example, RSFC with anterior and posterior DMN [97]. Resilience in young people was associated with increased gray matter volume in DMN regions such as MPFC and hippocampus [36], perhaps markers enhanced emotion and stress regulation ability [99]. Functional activity and connectivity in DMN is critically involved in whether individuals develop PTSD after trauma [100]. Increased structural and functional connectivity of DMN is a key feature differentiating disease expression and resilience between patients and their unaffected siblings in bipolar disorder [32], alcohol use disorder [101] and schizophrenia [102, 103]; DMN connectivity can be considered a protective feature that marks resilience [32, 102]. The present study extends this concept to protection against vicarious traumatization through enhanced capacity to cope with traumatic exposure via media [99].

This study has some limitations. First, MRI data were only acquired before COVID-19 pandemic, which did not allow us to describe the time-course of brain function and VT; longitudinal studies with repeated functional connectivity and behavior assessments would throw more light on mechanisms of brain-VT association. Second, VT and psychological resilience were measured using self-reported instruments, which combine self-beliefs, attitudes and values [104]; future studies should consider employing multiple methods including objective evaluations. Third, our subjects were all college students, which may limit the generalizability of our findings; studies are needed on populations with more diverse backgrounds (e.g., age, education, occupation, and mental illness). Forth, our study only found an association between VT and FCD in the ITG. It has been suggested that other DMN regions such as hippocampus, amygdala and MPFC are also involved in psychological trauma [105, 106]. Further research using other modalities (e.g., structural MRI) or analyses [107, 108] is needed to explore the relationship between VT and other DMN regions. Fifth, regions were identified based on a group-level atlas, and applying this to individual subjects may dilute brain-behavior associations [109]; future studies aimed at identifying individual-specific functional connectivity markers may capture these mechanisms more precisely. Finally, due to the exploratory nature of our study, caution is needed to interpret the current finding and the feasibility of our finding for use in clinical settings is limited and should be confirmed in future studies.

## Conclusion

Our study is the first to demonstrate the protective role of DMN functional connectivity against COVID-19 vicarious traumatization via psychological resilience. Our findings suggested that ITG and DMN might be a suitable target for the prevention and treatment of individuals at the risk of stress- and trauma-related mental disorders, e.g., by non-invasive brain stimulation (e.g., transcranial magnetic or direct current stimulation [110]), and have implications for the development of psychotherapy [111] aiming to foster psychological resilience in order to reduce the susceptibility to vicarious traumatization.

## Supplementary information

Supplementary Materials

## Data Availability

The data and code that support the findings of this study are available from the corresponding author through request. The data and code sharing adopted by the authors comply with the requirements of the funding institute and with institutional ethics approval.
