# Supplementary material for: Psychological resilience mediates the protective role of default-mode network functional connectivity against COVID-19 vicarious traumatization
Source: Transl Psychiatry. 2023 Jun 29;13:231. doi: 10.1038/s41398-023-02525-z (PMC10307857; doi:10.1038/s41398-023-02525-z)
Supplement: Supplementary file 1 — Supplementary Materials [file 41398_2023_2525_MOESM1_ESM.doc]

**Supplementary Tables**

**Table S1**. The normality of the scaled FCD in the whole-brain and major resting-state networks.

| Networks | K-S | P | S-W | P |
| --- | --- | --- | --- | --- |
| Whole-brain | 0.049 | 1 | 0.989 | 1 |
| Auditory network | 0.051 | 1 | 0.983 | 1 |
| Default-mode network | 0.087 | 0.522 | 0.975 | 0.450 |
| Dorsal attention network | 0.079 | 1 | 0.986 | 1 |
| Left central executive network | 0.075 | 1 | 0.962 | 0.054 |
| Right central executive network | 0.075 | 1 | 0.965 | 0.090 |
| Sensorimotor network | 0.086 | 0.585 | 0.975 | 0.522 |
| Ventral attention network | 0.073 | 1 | 0.976 | 0.594 |
| Visual network | 0.086 | 0.603 | 0.977 | 0.666 |

Note: The statistics were quantified by Kolmogorov–Smirnov (K-S) test and Shapiro-Wilk (S-W) test with Bonferroni correction for multiple comparisons.

**Supplementary Figures**


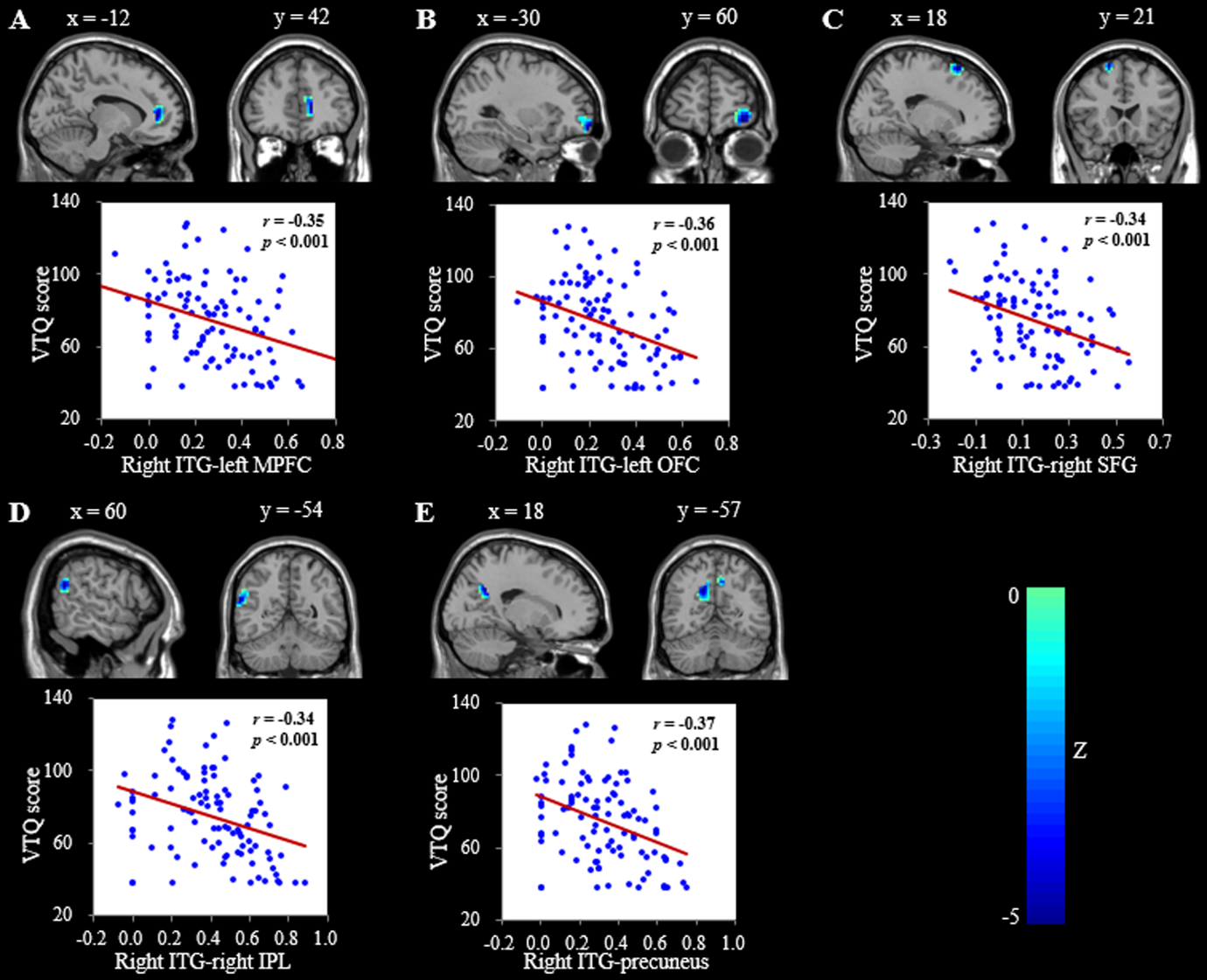


**Figure S1. Resting-state functional connectivity (RSFC) linked with vicarious traumatization.** Each panel (A) – (G) shows one of the brain regions whose functional connectivity strengths with the right ITG (seed) are linked to vicarious traumatization, and a scatter plot showing the correlation between vicarious traumatization and the functional connectivity strength of that brain region with right ITG. (Compared with Figure 2B, which shows the correlation between vicarious traumatization and the mean functional connectivity strength of all those brain regions with right ITG). Abbreviations: ITG, inferior temporal gyrus; MPFC, medial prefrontal cortex; OFC, orbitofrontal cortex; SFG, superior frontal gyrus; IPL, inferior parietal lobule; VTQ, Vicarious Traumatization Questionnaire.
